# Purification and Functional Characterization of the C-Terminal Domain of the β-Actin-Binding Protein AIM1 In Vitro

**DOI:** 10.3390/molecules23123281

**Published:** 2018-12-11

**Authors:** Fang Wu, Liangkai Cheng, Qi Yu, Lin Zhang, Hong Li, Caiyan Wang

**Affiliations:** Joint Laboratory for Translational Cancer Research of Chinese Medicine of the Ministry of Education of the People’s Republic of China, International Institute for Translational Chinese Medicine, Guangzhou University of Chinese Medicine, Guangzhou 510006, China; wf1003222@163.com (F.W.); chengliangkai88@163.com (L.C.); yuqi948226@163.com (Q.Y.); 15295786071@163.com (L.Z.); yaoxuexiatianli@163.com (H.L.)

**Keywords:** absent in melanoma 1 (AIM1), protein expression and purification, dimer, interactions between proteins

## Abstract

The protein absent in melanoma 1 (AIM1) is a member of the βγ-crystal lens superfamily that is associated with the development of multiple cancers. The binding of AIM1 to β-actin affects the migration and invasion of prostate cancer epithelial cells. The C-terminus of AIM1 is required for the β-actin interaction. However, the characteristics of AIM1 in vitro and the interaction mode between AIM1 and β-actin remain unknown. We describe novel methods to prepare pure recombinant AIM1 and identify possible binding modes between AIM1 and β-actin; we also obtain the crystal of the first two βγ-crystallin domains of AIM1 (g1g2) for future structural biology research. We first express and purify AIM1 after cloning the sequence into a modified pET-28a_psp expression vector. Next, we define the minimum unit formed by the βγ-crystallin domain repeats that bound to β-actin and perform its physiological function. Finally, we made the structural model of the AIM1 g1g2 that can be used to guide future biomedical investigations and prostate cancer research.

## 1. Introduction

More than 90% of cancers lead to deaths associated with metastasis. Cancer cells must have the ability to migrate into and invade surrounding tissues, then enter the lymphatic and blood microvasculature and eventually transfer to distant tissues and adapt to the environment to form a tumor [1]. Human cancer frequently shows morphological and molecular evidence of a dysregulated actin cytoskeleton.

A correlation between cytoskeletal dynamics and tumor development has been identified [2,3,4,5]. Photothermal therapy inhibits the collective migration of cancer cells by altering the cell structure and actin network [3,6]. The actin cytoskeleton is a dynamic cell scaffold that undergoes constant remodeling to promote structural plasticity and regulate cell motility, migration and invasion and is the major cytoskeleton in eukaryotic cells [7,8]. Its major structural component is β-actin. β-actin polymerizes in a helical fashion to form filaments (or microfilaments) that comprise the three-dimensional cytoskeletal network within eukaryotic cells, provides mechanical support and establishes the cell shape [9,10].

**Figure 1:**
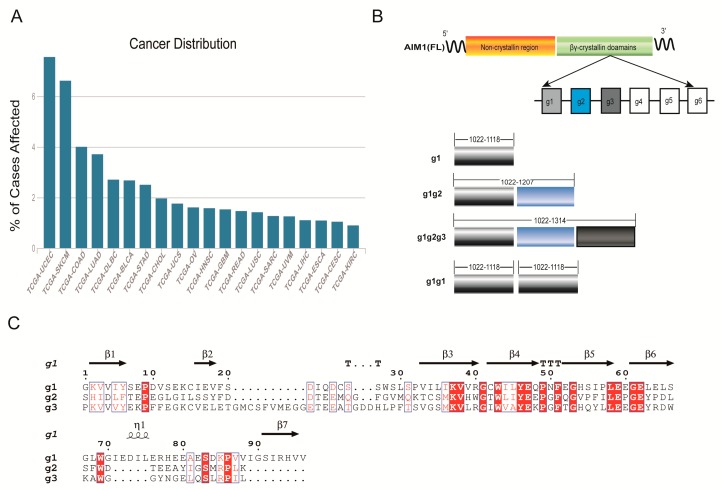
The relationship between AIM1 mutations and cancer and the division of AIM1 motifs. (**A**) Distribution of AIM1 mutants in various cancers. The cancers that are highly correlated with AIM1 mutations are mainly UCEC (Uterine Corpus Endometrial Carcinoma) and SKCM (Skin Cutaneous Melanoma). The number of cases examined was 338. Data were obtained from the TCGA database. (**B**) AIM1 domain partition map. In the figure, g1, g1g2, g1g2g3 and g1g1 are the topics of research in this study. (**C**) Sequence alignment of g1, g2, and g3. The red region is the more conserved part of the sequence.

The absent in melanoma 1 (AIM1) gene is a novel gene whose expression is correlated with tumor suppression in the human melanoma model [11,12,13]. Hypermethylation of the gene promoter is associated with the prognosis of some cancers [14]. According to an analysis of the Cancer Genome Atlas (TCGA) data, various AIM1 mutants are also associated with the development of cancer and have the greatest impact on some epithelial neoplasms and melanoma in the skin (Figure 1A). The gene is localized in chromosome 6q21, within the putative chromosome 6 tumor suppressor region for human melanoma [12]. Northern blot analyses revealed the highest expression of *AIM1* in adult skin, lung, heart, liver and kidney, and it plays a transient regulatory role in embryonic tissues [15]. The characterization of *AIM1* gene and its major transcripts reveal that AIM1 is a member of the βγ-crystallin superfamily [16]. Crystallin constitutes the major protein component of the vertebrate lens in the eye and the transparency of the lens is due to the high packing density of this protein [17]. The function of these proteins has not been clearly elucidated [18]. AIM1 contains 1723 amino acid residues, and the 1000-residue N-terminal region of AIM1 shows weak similarity to the neurofilament. AIM1 forms an elongated filament that interacts with the cytoskeleton, but further research has also revealed interactions of the C-terminus with the cytoskeleton. The C-terminus of βγ-crystallin domains contains six Greek motifs, comprising a total of 560 residues. The first domain of AIM1 is g1, the second domain of AIM1 is g2, the third domain of AIM1 is g3, and so on (Figure 1B). A sequence alignment of the amino acids of g1, g2, and g3 revealed a high degree of sequence conservation between domains (Figure 1C). One hundred thirty residues of the C-terminal region show local similarity to members of the gelsolin family. Gelsolins are actin regulatory proteins [17]. The first crystal structure of AIM1, “AIM1-g1”, confirmed that the protein is a calcium ion-dependent protein that can compete with other microbial homologs to bind calcium [19,20]. The second and third motifs of AIM1 have rarely been investigated and their functions remain unclear.

Previous studies show that AIM1 is a β-actin-binding protein that suppresses the invasion of prostate cancer cells. Depletion of AIM1 in prostate epithelial cells increases cytoskeletal remodeling, intracellular traction forces, cell migration and invasion, and cancer growth [7]. AIM1 tightly interacts with the actin cytoskeleton in prostate epithelial cells in normal tissues, and after deleting the C-terminal six Greek motifs, the protein lost the ability to bind to β-actin. However, a protein in which the last three Greek motifs were deleted retained the binding ability. To reveal the mechanism by which AIM1 binds to β-actin to inhibit the metastasis of malignant tumors, we explored which domain plays a major role in binding to β-actin.

We developed novel methods to prepare pure recombinant proteins and observe the AIM1 interaction with β-actin. For protein purification, we first performed preliminary purification with Ni-NTA, then removed the 8x His-tag and obtained the purified, unlabeled protein with a 1 mL HisTrap HP column. After separation on a 1 mL HiTrap QHP column, the pure protein was obtained, and finally, Superdex 200 increase 10/300GL resin was used to detect the aggregation state of the protein. g1, g1g2, g1g2g3 are dimers in solution. Dot blots revealed that g1g2 was the minimum unit that interacted with β-actin. In addition, we constructed an artificial recombinant protein containing two g1 repeats (named g1g1) and found that it also bound β-actin, further confirming that the first Greek motif g1 is required for the main physiological functions of AIM1.

## 2. Results

### 2.1. Modification of the Vector and Preparation of Prescission Protease

**Figure 2:**
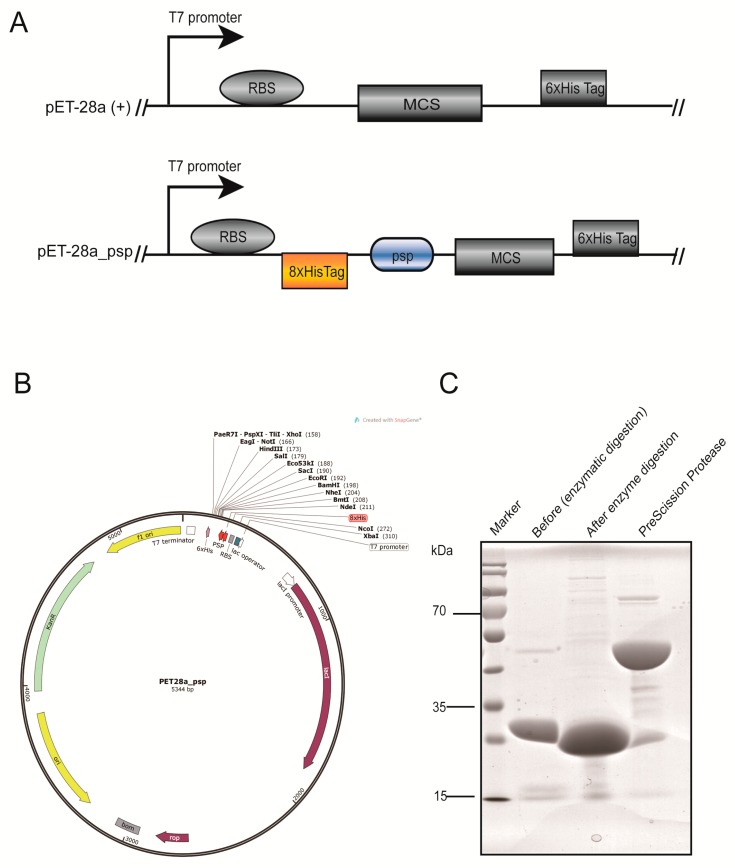
Construction of the pET-28a-psp plasmid and preparation of Prescission Protease. (**A**) The difference between the commercial pET-28a (+) and modified pET-28a_psp vectors. The orange and blue regions are the extra parts of the modified vector. RBS, Ribosomal Binding Site. MCS, Multiple Cloning Site. PSP, Prescission Proteas. (**B**) Map of the pET-28a_psp plasmid. The red region is the site recognized by the Prescission Protease and the position of the 8x His-tag. (**C**) The purity and activity of Prescission Protease were analyzed by SDS-PAGE (15%) and stained by Coomassie Blue.

We converted the commercially available pET-28a vector with a 6x His-tag to a pET-28a_psp vector with an 8x His-tag (Figure 2A,B). In our modified pET-28a_psp vector, the 8x His-tag is preceded by a cleavage site, followed by a multiple cloning site, so that the tag can be separated from the target protein by Prescission Protease recognition sequences. In addition, the 6x His-tag exists in commercial pET-28a (+) itself, can be terminated when the stop codon TGA was designed in the end of the target gene. Prescission Protease is expressed in the pGEX-6p1 vector and was purified on a Ni-NTA column. Purified Prescission Protease was used to remove the 8x His-tag. The extracted g1g2 and Prescission Protease were mixed at a ratio of 100:1 and incubated overnight. Purity of proteins were detected by SDS-PAGE gel (Figure 2C). The molecular weight of the g1g2 protein is 26.2 kDa and shifts to 23.8 kDa after treatment with Prescission Protease. The efficiency of the enzyme was approximately 95%. The purity of Prescission Protease was greater than 90% and the enzyme cleaved the 8x His-tag. The 8x His-tag was removed from the four proteins g1, g1g2, g1g2g3, and g1g1 using the same method.

### 2.2. The Cloning and Overexpression of g1, g1g2, g1g2g3, and g1g1

The coding sequences of these genes showed no variations or mutations from the T7 promotor to the stop codon. Plasmids containing the correct sequences were transformed into *Escherichia coli* and used for overexpression. The plasmids expressing g1, g1g2, g1g2g3 and g1g1 were constructed using the same method. *Escherichia coli* BL21 (DE3) cells harboring recombinant plasmids were cultured in LB medium. When the OD_600_ ranged between 0.6 and 0.8, 0.2 mM β-d-1-thiogalactopyranoside (IPTG) was added and cultured at 25 °C overnight. On SDS-PAGE gels, an overexpressed band was observed at the size of the target band.

### 2.3. Protein Extraction and Purification

**Figure 3:**
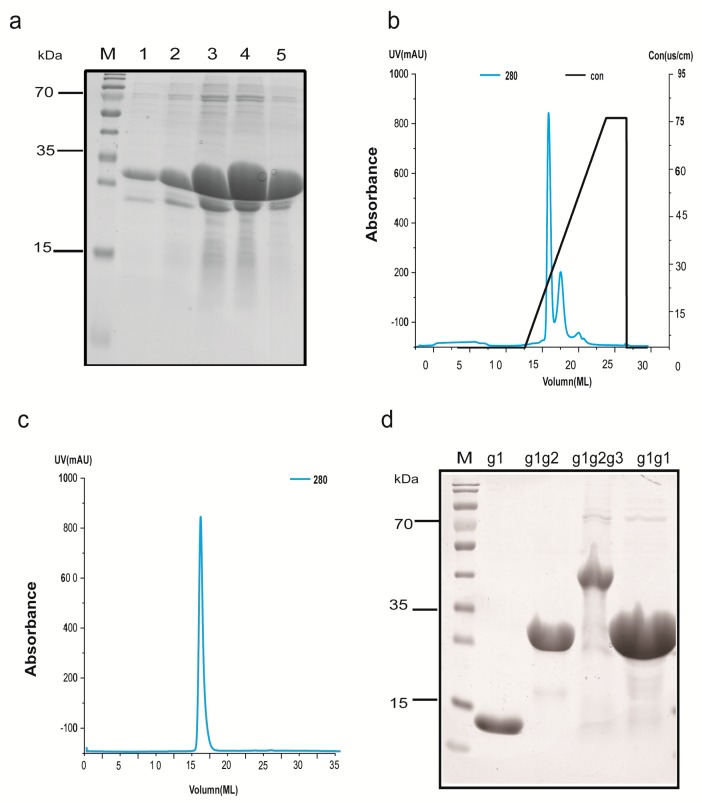
Purification procedures. (**a**) The sample is subjected to preliminary purification on a Ni-NTA column, and the purity was analyzed by 12% SDS-PAGE gels. M, molecular weight marker. Lanes 1–5, samples eluted sequentially at different times from the Ni-NTA column. (**b**) FPLC (1 mL HiTrap QHP) chromatogram showing effective separation. The blue line represents the UV absorption at 280 nM and the black line represents the change in the conductance. (**c**) Chromatogram of proteins purified with Superdex 200 increase 10/300GL. The blue line represents the UV absorption at 280 nM. (**d**) Analysis of the quality of the g1, g1g2, g1g2g3 and g1g1 proteins after purification on 12% SDS-PAGE gels.

This section describes g1g2 as an example. Resuspended cells were passed through a cryogenic overpressure cell breaker at 1200 psi. Both the supernatant and the precipitate were collected using high-pressure crushing followed by centrifugation. The recombinant soluble protein was purified on a Ni-NTA column (2 L of cells produce 15 mg of protein). Samples were analyzed by 12% SDS-PAGE and stained by Coomassie Blue (Figure 3a). Then, the ratio of Prescission Protease to protein was adjusted to 1:1000 and incubated overnight. The protein and 8x His-tag were separated using a 1 mL HisTrap HP column (the chromatogram of the 1 mL HisTrap HP column is not shown). The unbound fraction containing the target protein was collected. The 8x His-tag was not removed from the proteins used in the subsequent dot blot assay. An anion exchange chromatography QHP column was used to obtain the pure protein. The chromatogram of the 1 mL HiTrap QHP column showed two peaks (Figure 3b). The first peak (target protein peak) appeared at a volume of 15 mL and a conductivity of 22 ms/cm. The second peak (hetero protein peak) appeared at a volume of 17 mL and a conductivity of 28 ms/cm. Finally, the Superdex 200 increase 10/300GL resin was employed to obtain purified protein with better homogeneity for subsequent assays. Good protein homogeneity was achieved, because a single peak appeared at 16 mL (Figure 3c). The g1, g1g2, g1g2g3 and g1g1 proteins were purified using the same method. A single protein band appeared on the SDS-PAGE gel after purification and corresponded to the theoretical molecular weight of the fusion protein (Figure 3d). The molecular weight of g1 is 13.9 kDa, g1g2 is 26.23 kDa, g1g2g3 is 36.41 kDa and g1g1 is 25.58 kDa. The final yield of the soluble purified protein was 2 mg per liter of *Escherichia coli* culture and concentrate the protein to 3.5 mg/mL.

### 2.4. Characterization of AIM1 (g1, g1g2, g1g2g3, and g1g1) Formation in Solution

**Figure 4:**
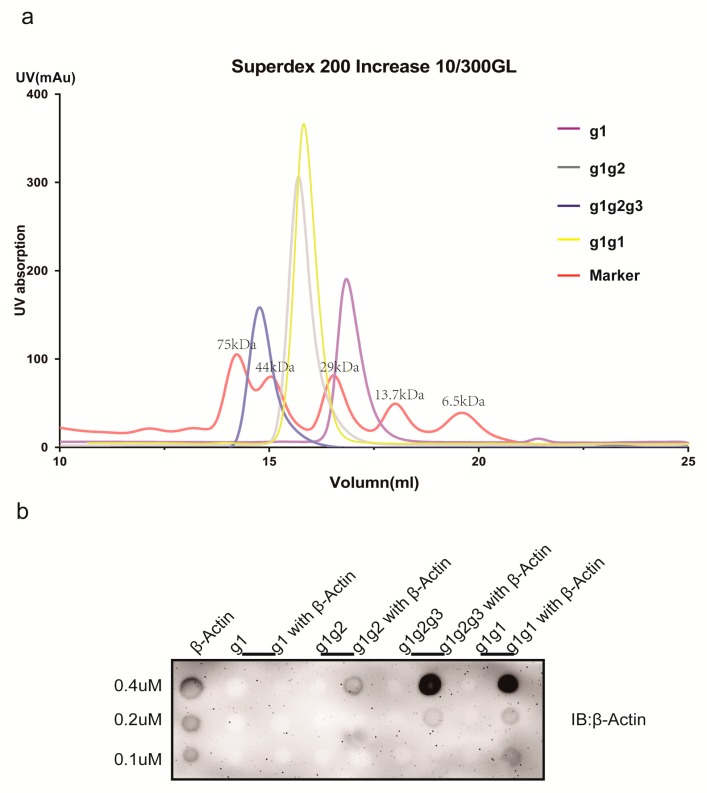
The aggregation of the protein in solution and binding to β-actin. (**a**) The chromatogram from a size-exclusion column showing the aggregation state of the protein. The red line indicates the molecular weight marker. The molecular weights from left to right are 75, 43, 29, 13.7 and 6.5 (kDa), respectively. (**b**) Dot blots of the interactions of g1, g1g2, g1g2g3, and g1g1 with β-actin. β-actin was derived from the total cell lysate of H1299 cells. The secondary antibody was a mouse anti-antibody.

The g1, g1g2, g1g2g3 and g1g1 proteins were applied to Superdex 200 increase 10/300GL resin to detect the aggregation in aqueous solution, and 1 mg of sample was assayed. Proteins were analyzed in the following Buffer: 150 mM NaCl, 20 mM Tris-HCl and 1 mM DTT. The chromatograms of the four proteins and the marker overlapped (Figure 4a). The g1 protein was located between the 13.7 kDa and 29 kDa markers, g1g2 and g1g1 were located between the 29 kDa and 43 kDa markers, g1g2g3 was located between the 43 kDa and 75 kDa markers, and the molecular weights of g1, g1g2, g1g2g3, and g1g1 are 13.9 kDa, 26.23 kDa, 36.41 kDa and 25.58 kDa, respectively. All of these proteins formed dimers in solution. According to previous studies, full-length g1g2g3 interacts with β-actin and g1g2 is a dimer in solution state. Therefore, we postulated that dimerization induced the formation of a ring in the three-dimensional structure, and the center of the ring is the binding pocket for β-actin. We further verified the assay to confirm this hypothesis.

### 2.5. Actin-Binding Assay

Dot blot assays were used to detect interactions between proteins. The control group and the experimental group included these four proteins incubated with β-actin. The proteins were loaded at concentrations of 0.4, 0.2 and 0.1 mM. The g1 did not bind to β-actin, while 0.4 mM g1g2 showed strong binding to β-actin. Interestingly, g1g1 also interacted with β-actin (Figure 4b). These findings further confirm that at least two motifs are required for the main physiological functions of AIM1. The assay was repeated more than three times.

### 2.6. Structural Model of g1g2 Interacting with G-Actin

**Figure 5:**
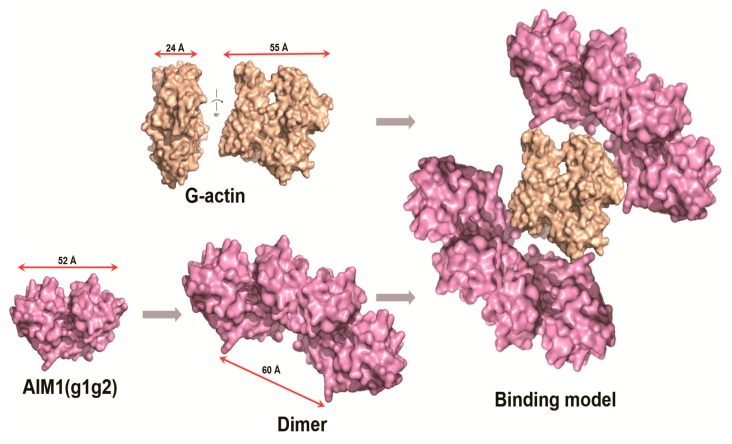
Structural model of g1g2 interacting with G-actin. The wheat color indicates G-actin. The magenta color represents g1g2. To obtain the structural model of the g1g2, we put the atomic structure of g1 from PDB (PDB ID: 3CW3) and the amino acid sequence of g1g2 into the website (www.sbg.bio.ic.ac.uk/phyre2page). The atomic structure of G-actin was got from PDB (PDB ID: 3HBT). The three-dimensional structure of g1g2 was predicted and the picture was prepared using PyMOL.

Based on the experimental results described above, we have modeled the interaction between g1g2 and G-actin (Figure 5). As shown in the figure, G-actin has a width of 24 Å and a length of 55 Å. It can exist as a monomer in solution [21]. For g1g2 to form a dimer, the interaction surface between the two monomers will be bent to a certain extent. Two g1g2 dimers can form a pocket with a diameter of approximately 60 Å. The size of the pocket precisely fits the width of G-actin to form an interaction surface, thereby further affecting the movement of the actin cytoskeleton and inhibiting the invasion and migration of the tumor cells.

## 3. Discussion

The production of soluble and active recombinant proteins is vital for structure-function analysis and therapeutic applications. Development of cancer drugs requires the ability to express and purify recombinant proteins, which are required in biological research to investigate enzyme activity, ligand binding, protein interactions, or other functions in vitro. The correlation between AIM1 and cancer has been reported in many studies [22,23,24], but researchers have not clearly determined whether this protein functions as a candidate tumor suppressor and its participation in cytoskeletal dynamics to inhibit tumor metastasis and invasion. Actin finishes its polymerization and depolymerization process by continuously transforming between G-actin in a monomeric state and F-actin in a fiber statete [25], subsequently affecting cell movement. The content of G-actin changes in invasive cancer cells, which is highly correlated with the phenotype of invasive cancer [26]. The content of G-actin in cells is regulated by AIM1 [7], and G-actin can exist as a monomer in solution [21]. We speculate that AIM1 is more likely to combine with G-actin. But further experimental verification is needed. Protein production is influenced by several factors, including the fusion tag, expression host, induction temperature and time. Our modified protocol for affinity purification exerted a positive effect on the purification of AIM1. Since some N-terminal His-tags might decrease the solubility of the protein, we obtained stable and purified recombinant AIM1 proteins by removing the 8x His-tag. This protocol might be useful for the purification of other similar proteins.

The first domain of AIM1 (which contains one motif β and γ) is named g1 in this paper and is very important for the stability and function of AIM1, according to the previous studies. In the present study, the first two domains of AIM1, g1g2, are the minimum unit required to bind β-actin. We designed a possible model for AIM1 g1g2 assembly with G-actin, as shown in Figure 5. Interestingly, g1g1 showed stronger binding ability than g1g2 to β-actin in dot blot experiments. g1 is the most divergent among βγ-crystallin in vertebrates [16,26], and it is most non-conservative motif in the six motifs of AIM1. During the biological evolution, it was found that the addition and deletion of some amino acids could obtain new functions [21], and some of the amino acid changes in g1 show its remarkable ability to adapt for novel functions [27]. Maybe this is the reason why g1g1 has stronger binding ability than g1g2 to β-actin. Also, many further experiments are needed for verification. The purity of the target protein was greater than 95%, and the protein displayed a single symmetric peak on the size-exclusion column. Following calibration with standard protein markers, the approximate protein size was calculated to be 44 kDa, suggesting that the protein formed a dimer in solution. AIM1 represents the most extreme case of this evolutionary process. It contains 12 βγ motifs arranged in an (βγ) 6 pattern. AIM1 g1g2 forms a dimer at the β-β interface. In this model, two AIM1 g1g2 dimers formed a pocket that precisely accommodates the width of G-actin to form an interaction surface. AIM1 domains might form intermolecular contacts instead of intramolecular contacts, perhaps by dimerizing with one or more additional AIM1 molecules or other βγ superfamily members.

Although AIM1 is a member of the βγ-crystallin family, its function is quite different, which is a hindrance to our further studies of its function. Currently, we only resolve the structure of the C-terminal g1 domain of this protein. The three-dimensional structure of the full-length protein need to be obtained to better understand its physiological functions; the crystallization of the protein is currently ongoing, and some crystallization procedures are being further optimized (data not shown). Based on our findings, at least two motifs are required for the physiological function of AIM1. This domain is only a very small part of the protein, and we are planning to perform further experiments.

## 4. Materials and Methods

### 4.1. Materials

*Escherichia coli* strains DH5α and BL21 (DE3) were the host strains for amplification and protein expression, respectively. pET-28a (+) was purchased from Novagen company. Subcloning of artificial genes encoding g1, g1g2, g1g2g3 and g1g1 were inserted into pET-28a_psp vectors and oligonucleotide primers were synthesized. The BamH1 and Sal1 restriction enzymes, T4 ligase and prestained protein ladder were purchased from Thermo Scientific. The Ni-NTA column, 1 mL HisTrap HP column, 1 mL HiTrap QHP column and Superdex 200 increase 10/300GL were purchased from GE Healthcare. AKTA pure 25 was purchased from GE Healthcare for protein purification. For dot blot assays, Super Enhanced ECL-chemiluminescence was purchased from sbjbio life science. All other Technologies from GBCBIOO chemicals used in the present study were of biochemical research grade.

### 4.2. Construction of the pET-28a_psp Vector

We have modified the commercial pET-28a (+) vector to facilitate the removal of the 8x His-tag. According to the base sequence of pET-28a (+), primers for pET-28a_psp were designed. The target fragment was obtained by PCR, purified, and treated with PNK enzyme to induce phosphorylation. After a second purification step, 50 ng of the system was brought to 50 mL and connected with T4 DNA ligase. The plasmid was transformed into the *Escherichia coli* DH5α strain, colonies were selected for sequencing and the correct sequences were confirmed. The final modified vectors contained an N-terminal 8x His-tag and with the Prescission Protease cleavage site (MGSSHHHHHHHHLEVLFQGP).

### 4.3. Cloning of AIM1 (g1, g1g2, g1g2g3, g1g1) into the pET-28a_psp Expression Vector

The g1, g1g2, and g1g2g3 proteins are AIM1 fragments of different lengths, and g1g1 is a repeating amino acid sequence of g1. The g1 fragment representing amino acids 1-97, g1g2 fragment representing amino acids 1-186 and g1g2g3 fragment representing amino acids 1-293 were artificially synthesized. The genes encoding g1, g1g2, g1g2g3 and g1g1 were amplified by PCR using five pairs of primers (Table 1).

These forward and reverse primers included the BamH1 and Sal1 restriction sites in pET-28a_psp. The pET-28a_psp plasmid was linearized by double digestion with the BamHI and SalI restriction enzymes and purified using a DNA Gel Extraction kit 50-Prep. The amplified PCR products and the linearized pET-28a_psp vector were ligated using DNA ligase. Finally, the recombinant plasmids were transformed into *Escherichia coli* BL21 (DE3) cells and spread on to an LB agar plate containing 30 μg/mL kanamycin. Colonies were evaluated using colony PCR.

### 4.4. Expression of AIM1 (g1, g1g2, g1g2g3, and g1g1) in Escherichia coli

The colonies containing correct sequences of the g1, g1g2, g1g2g3 and g1g1 cDNAs were grown in large-scale cultures in LB medium containing 30 μg/mL kanamycin at 37 °C. The cells were grown at 37 °C with shaking at 180 rpm until OD_600_ reached 0.6–0.8, and then protein expression was induced with 0.2 mM β-d-1-thiogalactopyranoside (IPTG) at 25 °C for 16 h. Cells were harvested by centrifugation (4000 rpm for 15 min at 4 °C) and stored at −80 °C until further use.

### 4.5. Protein Extraction and Purification

The previously collected strains were thawed and resuspended at in 40 mM Tris-HCl, pH 8.0, 250 mM sodium chloride, 1 mM β-mercaptoethanol and 1 mM PMSF (*w*/*v*). Resuspended cells were passed through cryogenic overpressure cell breaker at 1200 psi. The resulting lysate was centrifuged for 60 min at 12,000 rpm to obtain the supernatant. Firstly, the target protein was purified using a combination of affinity chromatography steps with Ni^2+^-nitrilotriacetic (Ni-NTA) agarose resin (GE Healthcare), and Buffer A (250 mM NaCl, 40 mM Tris-HCl, 10 mM imidazole) was used to remove hybrid proteins, followed by Buffer B (250 mM NaCl, 40 mM Tris-HCl, 250 mM imidazole) to elute the target protein. Secondly, the purification was performed by fast protein liquid chromatography (FPLC) on 1 mL HisTrap HP column (GE Healthcare, uppsala, Sweden) to obtain the target protein without 8x His-tag. The protein solution was injected into the equilibrated column at 0.5 mL/min flow rate, eluted using a linear imidazole gradient from 10 to 250 mM. Every 2 mL fraction was collected over the full gradient length. Thirdly, anion exchange chromatography was performed on a 1 mL QHP column (GE Healthcare) with NaCl gradient. Finally, the pure protein was separated on Superdex 200 increase 10/300GL (GE Healthcare) to obtain the homogeneous protein in solution. The column was first equilibrated with Buffer (150 mM NaCl, 40 mM Tris-HCl, 1 mM DTT), and the target proteins were concentrated. The aliquoted protein was flash-frozen and stored at −80 °C until further use.

### 4.6. Dot Blot Assay

The H1299 cells were lysed with RIPA Buffer for two hours and then centrifuged at high speed for 1 h, followed by quantification of the extracted total protein. The g1, g1g2, g1g2g3 and g1g1 proteins were extracted and purified using the method described above to finally obtain the 8x His-tagged proteins, and these proteins were incubated with TCL (Total Cell Lysate) at a 1:4 ratio of 1:4 for two hours on ice. The mixture was incubated with the appropriate amount of Ni-NTA for two hours at low temperature on a 3D shaker, and finally a Buffer containing imidazole was used to elute the desired protein. The proteins were quantified, spotted on PVDF membranes (unactivated), naturally air dried and blocked with 5% skim milk powder in TBST for 1 h. Membranes were incubated with a β-actin antibody overnight, washed, incubated with a secondary antibody, and exposed to ECL for detection.

## 5. Conclusions

We developed an effective procedure for the expression and isolation of milligram quantities of the highly purified six C-terminal Greek motifs of the AIM protein. The purified proteins were stable and formed a dimer in solution. The purified AIM1 g1g2 protein is the minimum unit required to bind β-actin. This protocol will be useful for further biochemical and structural biological studies.

## Figures and Tables

**Table 1:** Primers used for all recombinant plasmids mentioned in the text.

Gene Name	Primers
g1	F: GCGGCAGCGGATCCATGGGTAAAGTGGTTATCTACAGC R: TTGCACTTGTCGACTCAAACCACGTGACGAATGCTACC
g1g2	F: GCGGCAGCGGATCCATGGGTAAAGTGGTTATCTACAGC R: TTGCACTTGTCGACTCACTTCAGCGGACGCATGCTGCC
g1g2g3	F: GCGGCAGCGGATCCATGGGTAAAGTGGTTATCTACAGC R: TTGCACTTGTCGACTCACAGGATCGGGCGCAGGCTTTG
g1g1	F1: GCGGCAGCGGATCCATGGGTAAAGTGGTTATCTACAG R1: TTGCACTTGAATTCAACACGGTAGTCCTGAACCAC F2: GCGGCAGCGAATTCATGGGTAAAGTGGTTATCTACAGC R2: TTGCACTTGTCGACTCAAACCACGTGACGAATGCTACC
